# Supplementary material for: R2DT is a framework for predicting and visualising RNA secondary structure using templates
Source: Nat Commun. 2021 Jun 9;12:3494. doi: 10.1038/s41467-021-23555-5 (PMC8190129; doi:10.1038/s41467-021-23555-5)
Supplement: Supplementary file 1 — Supplementary Information [file 41467_2021_23555_MOESM1_ESM.pdf]

Supplementary Information

Supplementary Table 1. Newly developed R2DT templates using experimental data.

**rRNA type**	**Species**	**Clade**	**Supporting experimental data**
LSU	*Mycobacterium tuberculosis*	Bacteria (Actinobacteria)	5V7Q:A
LSU	*Staphylococcus aureus*	Bacteria (Firmicutes)	4WF9:X
LSU	*Tetrahymena thermophila*	Eukaryota	4V8P:D1+C2
LSU	*Bacillus subtilis*	Bacteria (Firmicutes)	5NJT:U
LSU	*Homo sapiens* mitochondria	Eukaryota	5OOL:A
LSU	*Homo sapiens*	Eukaryota	6EK0:L5+L8
LSU	*Dictyostelium discoideum*	Eukaryota	5AN9:N
LSU	*Thermus thermophilus*	Bacteria (Deinococcus-Thermus)	4Y4O:2A
LSU	*Haloarcula marismortui*	Archaea	4V9F:0
LSU	*Mycobacterium smegmatis*	Bacteria (Actinobacteria)	5O60:A
LSU	*Plasmodium falciparum*	Eukaryota	3J79:A+C
LSU	*Saccharomyces cerevisiae*	Eukaryota	5TBW:1+4
LSU	*Spinacia oleracea* chloroplast	Eukaryota	6ERI:AA
LSU	*Escherichia coli*	Bacteria (Gammaproteobacteria)	5J7L:DA
LSU	*Deinococcus radiodurans*	Bacteria (Deinococcus-Thermus)	4IOA:X
LSU	*Drosophila melanogaster*	Eukaryota	4V6W:A5
LSU	*Kluyveromyces lactis*	Eukaryota	4V91:1+4

LSU	Lokiarcheaota F3H4	Archaea	Based on [1]
LSU	Lokiarcheaota GC1475	Archaea	Based on [1]
LSU	*Tetrahymena thermophila* mitochondria	Eukaryota	Based on [1]
SSU	*Homo sapiens*	Eukaryota	4V6X:B2
SSU	*Thermus thermophilus*	Bacteria (Deinococcus-Thermus)	4V51:AA
SSU	*Pyrococcus furiosus*	Archaea	4V6U:A2
SSU	*Drosophila melanogaster*	Eukaryota	4V6W:B2
SSU	*Saccharomyces cerevisiae*	Eukaryota	4V88:A2
SSU	*Trypanosoma brucei*	Eukaryota	4V8M:AA
SSU	*Escherichia coli*	Bacteria (Gammaproteobacteria)	4V9D:AA
RNAse P	*Methanocaldococcus jannaschii*	Archaea	6K0A:A
RNAse P	*Bacillus stearothermophilus*	Bacteria	2A64:A
RNAse P	*Thermotoga maritima*	Bacteria	3Q1R:A
RNAse P	*Homo sapiens*	Eukaryota	6AHR:A

Supplementary Table 2. Summary of template to diagram similarity by phylogenetic rank for RefSeq rRNA sequences.

Taxonomic Rank	Number of nucleotides positioned exactly as in template	Number of nucleotides inserted compared to template	Number of nucleotides requiring repositioning compared to template	Total number of displayed nucleotides	Number of diagrams
species	259,034 (97.%)	4,175 (1.6%)	3,896 (1.5%)	267,105	147 (.6%)
genus	538,357 (98.%)	5,759 (1.%)	5,332 (1.%)	549,448	384 (1.6%)
family	868,585 (98.%)	9,279 (1.%)	8,629 (1.%)	886,493	558 (2.3%)
order	1,390,705 (98.%)	15,281 (1.1%)	13,761 (1.%)	1,419,747	860 (3.6%)
class	6,264,956 (97.5%)	68,719 (1.1%)	89,065 (1.4%)	6,422,740	3,844 (16.1%)
phylum	7,094,429 (97.3%)	104,029 (1.4%)	91,687 (1.3%)	7,290,145	4,780 (20.%)
kingdom	20,172,007 (95.4%)	557,422 (2.6%)	420,121 (2.%)	21,149,550	13,224 (55.5%)
superkingdom	28,360 (94.2%)	757 (2.5%)	997 (3.3%)	30,114	8 (.%)
root	20,410 (37.5%)	838 (1.5%)	33,142 (60.9%)	54,390	38 (.2%)
**Total**	**36,636,843 (96.2%)**	**766,259 (2.%)**	**666,630 (1.8%)**	**38,069,732**	**23,843**

Supplementary Table 3. Sample of R2DT run times for a range of sequences. Measurements were taken on a CentOS machine with 10 GB of RAM and an Intel(R) Xeon(R) Gold 6252 CPU at 2.10 GHz.

PDB ID	RNAcentral identifier	Sequence length	RNA type	R2DT run time (m:ss)
6AHR Chain A	URS000013F331_9606	417	*Homo sapiens* RNase P	0:10
4V4Q Chain AA	URS00000ABFE9_562	1,542	*Escherichia coli* SSU rRNA	0:15
6G4W Chain 2	URS0000D56C31_9606	1,882	*Homo sapiens* SSU rRNA	0:15
6RXV Chain C2	URS0000EEACFC_209285	230	*Chaetomium thermophilum* U3 snoRNA	0:18
3J9M Chain A	URS000080E357_9606	1,559	*Homo sapiens* mitochondrial SSU rRNA	0:41
5W4K Chain 1w	URS00005AA258_562	76	*Escherichia coli* tRNA	0:45
6EK0 Chain L7	URS000002B0D5_9606	120	*Homo sapiens* 5S rRNA	0:46
4V4Q Chain BB	URS00004B0F34_562	2,904	*Escherichia coli* LSU rRNA	0:49
4UG0 Chain L5	URS000086853A_9606	5,070	*Homo sapiens* LSU rRNA	1:47

References

1. Penev, P. I. *et al.* Supersized ribosomal RNA expansion segments in Asgard archaea. doi:10.1101/2019.12.25.888164.
