# Supplementary material for: R2DT is a framework for predicting and visualising RNA secondary structure using templates
Source: Nat Commun. 2021 Jun 9;12:3494. doi: 10.1038/s41467-021-23555-5 (PMC8190129; doi:10.1038/s41467-021-23555-5)
Supplement: Supplementary file 3 — Description of Additional Supplementary Files [file 41467_2021_23555_MOESM3_ESM.pdf]

Description of Additional Supplementary Files

File Name: Supplementary Data 1
Description: The dataset contains 1,043 RNA secondary structure diagrams generated by R2DT based on the HGNC and EcoCyC sequences from human and Escherichia coli, respectively. The images have been manually inspected to identify any modes of failure and have been classified into accepted (97.7%) and rejected (2.3%) diagrams.
