# Supplementary material for: R2DT is a framework for predicting and visualising RNA secondary structure using templates
Source: Nat Commun. 2021 Jun 9;12:3494. doi: 10.1038/s41467-021-23555-5 (PMC8190129; doi:10.1038/s41467-021-23555-5)
Supplement: Supplementary file 5 — Reporting Summary [file 41467_2021_23555_MOESM5_ESM.pdf]

nature research

Corresponding author(s): Drs Anton I. Petrov and Robert D. Finn

Last updated by author(s): 29/04/2021

# Reporting Summary

Nature Research wishes to improve the reproducibility of the work that we publish. This form provides structure for consistency and transparency in reporting. For further information on Nature Research policies, see our Editorial Policies and the Editorial Policy Checklist.

## Statistics

For all statistical analyses, confirm that the following items are present in the figure legend, table legend, main text, or Methods section.

| n/a | Confirmed | |
|---|---|---|
| ☒ | ☐ | The exact sample size (*n*) for each experimental group/condition, given as a discrete number and unit of measurement |
| ☒ | ☐ | A statement on whether measurements were taken from distinct samples or whether the same sample was measured repeatedly |
| ☒ | ☐ | The statistical test(s) used AND whether they are one- or two-sided<br>*Only common tests should be described solely by name; describe more complex techniques in the Methods section.* |
| ☒ | ☐ | A description of all covariates tested |
| ☒ | ☐ | A description of any assumptions or corrections, such as tests of normality and adjustment for multiple comparisons |
| ☒ | ☐ | A full description of the statistical parameters including central tendency (e.g. means) or other basic estimates (e.g. regression coefficient) AND variation (e.g. standard deviation) or associated estimates of uncertainty (e.g. confidence intervals) |
| ☒ | ☐ | For null hypothesis testing, the test statistic (e.g. *F*, *t*, *r*) with confidence intervals, effect sizes, degrees of freedom and *P* value noted<br>*Give P values as exact values whenever suitable.* |
| ☒ | ☐ | For Bayesian analysis, information on the choice of priors and Markov chain Monte Carlo settings |
| ☒ | ☐ | For hierarchical and complex designs, identification of the appropriate level for tests and full reporting of outcomes |
| ☒ | ☐ | Estimates of effect sizes (e.g. Cohen's *d*, Pearson's *r*), indicating how they were calculated |

*Our web collection on statistics for biologists contains articles on many of the points above.*

## Software and code

Policy information about availability of computer code

| | |
|---|---|
| Data collection | All data were produced and analysed using the R2DT software (v1.1). The R2DT source code is available on GitHub under the Apache 2.0 License (https://github.com/rnacentral/R2DT). An R2DT web server can be found at https://rnacentral.org/r2dt and its source code is available at https://github.com/RNAcentral/r2dt-web. The following tools have also been used in this study: tRNAscan-SE 2.0.5, VARNA v3.9, RNA2Drawer 2.0, Infernal v1.1.2, Ribovore 0.40 , Traveler v2.0, R2DT v1.1, Forna 1.0, PseudoViewer v3.0, and XRNA-GT 1.1. |
| Data analysis | In addition, we created a custom version of the XRNA software called XRNA-GT (v1.1) which is available at https://github.com/LDWLab/XRNA-GT. XRNA-GT can be used to analyse and improve individual secondary structures produced by R2DT. |

For manuscripts utilizing custom algorithms or software that are central to the research but not yet described in published literature, software must be made available to editors and reviewers. We strongly encourage code deposition in a community repository (e.g. GitHub). See the Nature Research guidelines for submitting code & software for further information.

## Data

Policy information about availability of data

All manuscripts must include a data availability statement. This statement should provide the following information, where applicable:

- Accession codes, unique identifiers, or web links for publicly available datasets
- A list of figures that have associated raw data
- A description of any restrictions on data availability

The set of precomputed RNAcentral 2D structures are available at https://rnacentral.org. The diagrams are continuously updated as additional templates are developed or algorithm improvements are made. The sequences used in this study were obtained from RNAcentral release 15 and EcoCyc release 24.0.

# Field-specific reporting

Please select the one below that is the best fit for your research. If you are not sure, read the appropriate sections before making your selection.

☒ Life sciences ☐ Behavioural & social sciences ☐ Ecological, evolutionary & environmental sciences

For a reference copy of the document with all sections, see nature.com/documents/nr-reporting-summary-flat.pdf

# Life sciences study design

All studies must disclose on these points even when the disclosure is negative.

| | |
|---|---|
| Sample size | In the course of the study we analyzed 16,107,505 sequences from the RNAcentral release 15. |
| Data exclusions | No data was excluded from the analysis as the entire RNAcentral dataset has been analyzed. |
| Replication | The R2DT algorithm is deterministic so no replication was needed. |
| Randomization | As the algorithm is deterministic, randomization was not used. |
| Blinding | No statistical analysis was performed so blinding was not required. |

# Reporting for specific materials, systems and methods

We require information from authors about some types of materials, experimental systems and methods used in many studies. Here, indicate whether each material, system or method listed is relevant to your study. If you are not sure if a list item applies to your research, read the appropriate section before selecting a response.

## Materials & experimental systems

| n/a | Involved in the study | |
|---|---|---|
| ☒ | ☐ | Antibodies |
| ☒ | ☐ | Eukaryotic cell lines |
| ☒ | ☐ | Palaeontology and archaeology |
| ☒ | ☐ | Animals and other organisms |
| ☒ | ☐ | Human research participants |
| ☒ | ☐ | Clinical data |
| ☒ | ☐ | Dual use research of concern |

## Methods

| n/a | Involved in the study | |
|---|---|---|
| ☒ | ☐ | ChIP-seq |
| ☒ | ☐ | Flow cytometry |
| ☒ | ☐ | MRI-based neuroimaging |
